# To Fall or Not to Fall – Falls and Perceptions of Control and Meaning of the Home

**DOI:** 10.1093/geroni/igab046.1716

**Published:** 2021-12-17

**Authors:** Björn Slaug, Susanne Iwarsson, Steven Schmidt

**Affiliations:** Lund University, Lund, Skane Lan, Sweden

## Abstract

Falls are a major public health problem among older people. Even if the outcome of a fall is not fatal, it may be a traumatic experience with both physical and psychological consequences. However, there is a lack of studies examining how falls in the home may impact the perception of the home. To compare perceptions of the home between those who had fallen and those who had not, we utilized data from the Swedish SNAC-GÅS study (N=371; mean age=68; 43% men). Perceptions of control and meaning of the home were captured by established psychological instruments. Excluding falls in other environments than the home (n=99), those who fell in the home the previous year (n=34) scored notably higher on housing control by “powerful others” (p=0.053) and notably lower on perceived “behavioral home bonding” (p=0.056) compared to non-fallers (n=238). These results warrant further research into the impact of falls on perceived housing.

